# Disproportionality in Power Plants’ Carbon Emissions: A Cross-National Study

**DOI:** 10.1038/srep28661

**Published:** 2016-07-01

**Authors:** Andrew Jorgenson, Wesley Longhofer, Don Grant

**Affiliations:** 1 Department of Sociology and the Environmental Studies Program, Boston College, 140 Commonwealth Avenue, Chestnut Hill, MA 02467 USA; 2 Goizueta Business School, Emory University, 1300 Clifton Road, Atlanta, GA 30322 USA; 3 Department of Sociology and the Renewable and Sustainable Energy Institute, University of Colorado at Boulder, Boulder, CO 80309 USA

## Abstract

Past research on the disproportionality of pollution suggests a small subset of a sector’s facilities often produces the lion’s share of toxic emissions. Here we extend this idea to the world’s electricity sectors by calculating national-level disproportionality Gini coefficients for plant-level carbon emissions in 161 nations based on data from 19,941 fossil-fuel burning power plants. We also evaluate if disproportionalities in plant-level emissions are associated with increased national carbon emissions from fossil-fuel based electricity production, while accounting for other well-established human drivers of greenhouse gas emissions. Results suggest that one potential pathway to decreasing nations’ greenhouse gas emissions could involve reducing disproportionality among fossil-fuel power plants by targeting those plants in the upper end of the distribution that burn fuels more inefficiently to produce electricity.

The combustion of fossil fuels for the production of electricity comprises the single largest contributor to sectoral-level anthropogenic greenhouse gases, accounting for roughly a quarter of all emissions^1^,^2^. The Intergovernmental Panel on Climate Change recently suggested that carbon emissions from the energy supply sector could double and perhaps triple from 2010 baseline levels by 2050^3^, given the growth in the number of power plants throughout the world, especially in rapidly developing nations where fossil-fuel burning power plants make up a large portion of the energy sector^4^. Thus, in order to meet the goals set in the Paris climate accord, countries should explore policy strategies that address the disproportionate contributions of the energy supply sector to overall emissions.

Also important is disproportionate emissions within a nation’s energy supply sector. Recent preliminary research by Grant and colleagues^5^ examined power plants in 20 nations that account for the majority of the world’s electricity-based carbon emissions. Through a descriptive analysis of the distributions of plant-level emissions and their emissions intensities, it was found that the dirtiest 5 percent of power plants are responsible for substantial shares of their nation’s emissions from electricity generation. If these plants continued generating the same amount of electricity but did so at average intensities or levels of efficiency relative to other plants within the same nation, the world’s total electricity-based carbon emissions could be reduced by as much as 40 percent^5^.

The results of Grant and colleagues^5^ preliminary study on power plants is consistent with Freudenburg’s^6^ pioneering sociological research on disproportionality in the production of environmental pollution among manufacturing facilities within the United States. Freudenburg hypothesized that a small subset of a sector’s facilities is often responsible for the lion’s share of its toxic emissions. Using data from the Toxic Release Inventory, Freudenburg calculated Gini coefficients to quantify disproportionality in facility-level pollution. The Gini coefficient is a widely used measure of inequality among values within a frequency distribution, and is most often employed in research on income and wealth inequities. In particular, the Gini coefficient measures the area between the Lorenz curve and the hypothetical line of absolute equality, expressed as a percentage of the maximum area under the line. The values of estimated Gini coefficients can range from zero (perfect equality, perfect proportionate distribution) to 100 (perfect inequality, perfect disproportionate distribution).

Freudenburg^6^ found substantial inequality in emissions, and such disproportionalities in toxic releases were amplified when industries were normalized by size, suggesting that varying levels of pollution are not simply the result of higher levels of production in some industries versus others. Freudenburg concluded that toxic pollution within the United States could be mitigated significantly if a relatively small fraction of producers substantially increased their efficiency and that such improvements would not greatly disrupt the overall economy or threaten industry survival. Freudenberg’s approach to analyzing disproportionality in the production of pollution/emissions has been adopted and expanded by other researchers in recent years, including in studies that link industrial pollution disproportionalities to public health disparities and environmental justice communities within the United States^7^,^8^.

In this study we employ Freudenburg’s^6^ approach to examine disproportionalities in the emissions of fossil-fuel power plants for the majority of the world’s nations. Using facility-level data for 19,941 fossil-fuel plants throughout the globe, which we obtained from the Center for Global Development’s Carbon Monitoring for Action data file (see Methods Section for details on all data used in the analysis), we estimate national-level Gini coefficients for 161 nations for the year 2009, the most recent year in which these data are currently available. The 161 nations are those where the plants within the overall sample are located. We weight each plant by plant-level output, measured as net megawatt hours generated in 2009, thereby taking into account in the calculation of the national Gini coefficients how efficiently electricity is generated for each fossil-fuel power plant.

Besides being the first study to quantify such disproportionalities in power plant carbon emissions for the majority of the world’s nations, we also evaluate if disproportionalities in plant-level emissions are associated with nations’ overall carbon emissions from fossil-fuel based electricity production, while controlling for other well-established human drivers of greenhouse gas emissions^9^,^10^,^11^. If it is found that national-level emissions from fossil-fuel power plants are associated with disproportionality in plant-level emissions, especially after accounting for the effects of the established human drivers, then targeting extreme polluters in the electricity generation sector to become more efficient could be a viable climate change mitigation strategy for both developed and developing nations.

## Results

**Figure 1:**
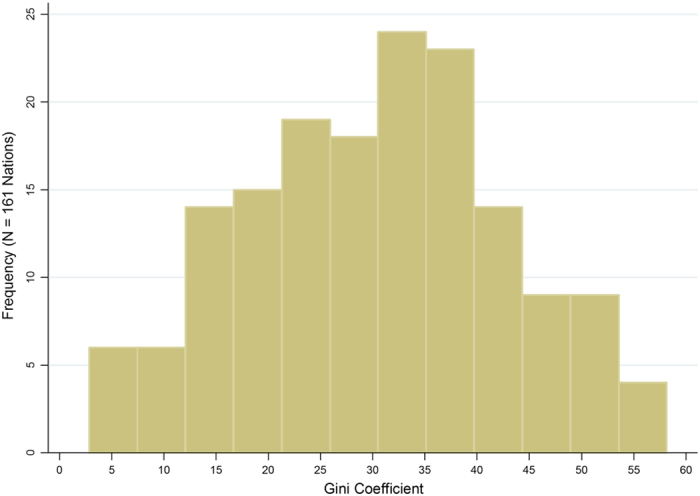
Histogram of the Gini Coefficients for Disproportionality in Plant-Level Carbon Emissions for 161 Nations, 2009.

Figure 1 is a histogram for the estimated disproportionality Gini coefficients for the 161 nations in the study. With a skewness value of −0.03, the distribution of the Gini coefficients is very close to normal. The disproportionality Gini coefficients have a mean of 30.38, a median of 31.17, and a standard deviation of 12.58. The values of the Gini coefficients range from a low of 2.85 (Namibia) to a high of 58.17 (Germany). The descriptive statistics indicate a notable amount of variation in disproportionality in plant-level carbon emissions across nations as well as a relatively symmetrical distribution. More importantly, all nations in the sample are characterized by disproportionality to some extent in their fossil-fuel power plants’ carbon emissions.

Table 1 lists the disproportionality Gini coefficients for each of the 20 nations with the largest national carbon emissions from such power plants for the year 2009. These 20 nations accounted for slightly more than 86% of the total emissions from fossil-fuel power plants for the full sample of 161 nations for the same year.

Table 1 also lists the number of fossil-fuel plants within each of these nations as well as the percent of nations’ fossil-fuel plants who’s primary fuel are coal, gas fossil-fuels, and liquid fossil-fuels. (For similar information on all nations in the study, see Supplementary Table 1). This additional information is provided so it can be determined if national-level disproportionality in power plant emissions is associated with the actual number of such plants within nations as well as the composition of fossil fuels being used by plants within nations.

China, a rapidly developing nation with the highest national-level emissions from fossil-fuel power plants, has a Gini coefficient of 35.87 for 1130 fossil-fuel plants, 81.50 percent of which burn coal as a primary fuel. The United States, the second biggest national emitter, has a larger Gini coefficient of 48.86 and for more than twice as many fossil-fuel power plants (2612 plants) than for China, for which 53.52 percent use gas fossil-fuels as their primary fuel source.

India and Russia, both large developing nations ranked third and fourth in national emissions, have Gini coefficients relatively similar in value as the United States, but with a fraction of the same number of plants (737 and 529). For India’s plants, 43.42 percent burn liquid fossil-fuels as their primary fuel, 37.72 percent burn coal, and 18.86 percent burn gas fossil-fuels. However, 65.97 percent of Russia’s plants burn gas fossil-fuels as their primary fuel, with the remaining split between coal (19.28 percent) and liquid fossil-fuels (14.75 percent).

Kazakhstan and Thailand, developing nations ranked 19^th^ and 20^th^ in national emissions from 51 and 92 fossil-fuel power plants, have Gini coefficients of 50.68 and 45.03. The majority of Kazakhstan’s plants burn coal (52.94 percent), while the majority of Thailand’s plants burn gas fossil-fuels (54.35 percent) as their primary fuel.

For the 20 highest national emitters, which consist of both developed and developing nations, the associations between disproportionality in plant-level carbon emissions and the number of plants within a nation as well as the percent of nations’ plants that primarily burn coal, gas fossil-fuels, or liquid fossil-fuels are relatively weak. We note that none of the Pearson’s correlation coefficients for these associations are statistically significant, and none are larger than 0.1 in absolute value. The lack of statistical significance is likely due to the small sample size (i.e., N = 20).

For all 161 nations in the study, the bivariate associations (i.e., Pearson’s correlation coefficients) are relatively stronger. The national-level Gini coefficients for disproportionality in plant-level emissions in 2009 are correlated at 0.42 with the number of fossil-fuel plants within a nation, 0.13 with the percent of nations’ plants whose primary fuel is coal, 0.27 with the percent of nations’ plants that primarily burn gas fossil-fuels, −0.30 with percent nations’ plants that use liquid fossil-fuels as their primary fuel, 0.17 with Gross Domestic Product per capita (measured in 2005 U.S. Dollars), and 0.22 with national-level carbon emissions from fossil-fuel power plants. All of these correlations are statistically significant at the 0.05 level (two-tailed tests), with the exception of the correlation between the disproportionality Gini coefficient and percent of nations’ plants that burn coal as their primary fuel. The latter correlation is statistically significant at the 0.10 level (two-tailed test).

Next, we conduct a cross-sectional regression analysis to estimate the effect of the disproportionality in power plant emissions within nations on national-level carbon emissions from fossil-fuel power plants for the year 2009, while taking into account the effects of well-established human drivers of national-level emissions^9^. We include measures of population size, gross domestic product per capita, and trade as a percent of gross domestic product. Population size and gross domestic product per capita are the two most commonly assessed human drivers of national carbon emissions and together tend to explain a large proportion of variation in emissions across nations^9^. Trade as a percent of gross domestic product is a commonly used measure of nations’ relative levels of integration in the world economy, and the effects of world-economic integration has been the focus of much recent sociological research on the human drivers of national greenhouse gas emissions^10^.

We also control for the number of fossil-fuel power plants within a nation as well as whether or not a nation is located in a tropical climate, the average price of electricity for each nation, and the percent of a nation’s fossil-fuel power plants who’s primary fuel are (1) coal, (2) gas fossil-fuels, and (3) liquid fossil-fuels. Recent research indicates that these factors are associated with carbon emissions from fossil-fuel power plants^11^ (See Methods Section for details on all data and their sources).

We estimated the models with both ordinary least squares (OLS) regression and robust regression, and consistent with past research on the drivers of anthropogenic greenhouse gas emissions^9^,^12^,^13^, all non-binary variables were converted into logarithmic form (base 10), leading to the estimation of elasticity coefficients. The elasticity coefficient for an independent variable is the estimated net percentage change in the dependent variable associated with a 1 percent increase in the independent variable. (See Methods Section for additional details on the model estimation techniques).

The results of the regression analysis are provided in Table 2 (models 1–4) and Table 3 (models 5–7). We report 7 OLS and robust regression models to illustrate the consistency in the statistically significant effect of the Gini coefficient for disproportionality in plant-level emissions on national-level carbon emissions across models with different control variables. Since (1) we are investigating if national emissions are positively associated with the disproportionality Gini coefficient, and (2) the sample size for the cross-sectional regression analysis is relatively small (i.e., 161 nations), we conduct one-tailed tests of statistical significance.

The elasticity coefficient for the Gini coefficient in the OLS models ranges from 0.54 to 0.70. For these models, a 1 percent increase in disproportionality in plant-level carbon emissions leads to, at minimum, a 0.54 percent increase in national-level carbon emissions. For the robust regression models, the estimated effect (elasticity coefficient) for the Gini coefficient ranges in value from 0.37 to 0.47. Robust regression is a statistical modeling approach that down-weights the influence of influential cases, sometimes leading to the estimation of coefficients that are relatively more conservative than those derived from OLS regression^14^. These findings suggest that a 1 percent increase in disproportionality in plant-level emissions leads to at least a 0.37 percent increase in national-level carbon emissions from fossil-fuel power plants.

Turning to the control variables, both population size and gross domestic product per capita exhibit positive and statistically significant effects on national emissions and with marginal differences across the OLS and robust regression models. The estimated effect of the number of fossil-fuel power plants is nonsignificant in all but two models where it achieves a marginal level of statistical significance, and its inclusion appears to slightly suppress the estimated effect of the Gini coefficient for disproportionality in the OLS models. However, when the number of fossil-fuel power plants is added as a control in the robust regression models, the estimated effect of the Gini coefficient on national emissions moderately increases in value. Initially, the coefficient for tropical climate is negative and statistically significant, but becomes nonsignificant once the controls for percent of nations’ plants using different primary fossil-fuel sources are added to the models.

The elasticity coefficient for average price of electricity is negative and statistically significant across multiple OLS and robust regression models. Conversely, the elasticity coefficient for the percent of nations’ fossil-fuel power plants that use coal as a primary fuel source is positive and statistically significant across all relevant models. The estimated effect of the percent of nations’ plants that primarily use gas fossil-fuels is positive and statistically significant in the robust regression models, but nonsignificant in the OLS models, while the elasticity coefficients for the percent of nations’ plants that primarily use liquid fossil-fuels are all nonsignificant. The estimated effect of trade as a percent of gross domestic product is positive, marginally statistically significant, and very similar in value for the OLS and robust regression models. Including these controls only slightly suppresses the estimated effect of disproportionality in plant-level emissions on national carbon emissions.

## Discussion

Our study is the first to apply Freudenburg’s^6^ disproportionality approach to the majority of the world’s fossil-fuel power plants. We find that countries vary significantly in their level of disproportionality, and all nations exhibit some level of disproportionality in power plant emissions. Further, our cross-national regression analysis suggests a positive association between national-level carbon emissions from fossil-fuel power plants and disproportionality in plant-level emissions within nations, even after accounting for the most well-established human drivers of national carbon emissions. In particular, the results suggest that a 1 percent increase in disproportionality in plant-level emissions leads to, at minimum, a 0.37 percent increase in national emissions from fossil-fuel power plants. We suggest this is a non-trivial association that is worthy of scholarly attention and additional research.

The results raise an important policy implication: to reduce the contributions of the electricity sector to overall greenhouse gas emissions in order to meet goals established in the recent Paris accord, some nations should consider reducing disproportionality among their fossil-fuel power plants by targeting a modest number of plants in the upper end of the distribution that burn fuels less efficiently. Policies that require or incentivize power plants in the upper tail of the distribution to produce electricity more efficiently could potentially go a long way toward reducing the sector’s contributions to climate change, consistent with the implications of prior preliminary research^5^, but inconsistent with many previous policy proposals in the United States and elsewhere^15^,^16^,^17^. However, just as deliberations over the Paris accord were marked by observations of differentiated responsibility, no disproportionality policy fits all. In countries where larger emitters are harder to regulate, pursuing other policy strategies, such as targeting a greater number of smaller emitters, may be more effective. In any case, this research suggests that at minimum policies targeting extreme polluters at the facility level should be considered alongside those that focus on sector-wide characteristics and systemic conditions to reduce anthropogenic emissions.

Our Gini coefficients for disproportionality in plant-level carbon emissions do not tell the whole story. While a Gini coefficient does provide a single snapshot of how emissions are distributed among fossil-fuel power plants in a given nation, it does not by itself indicate which power plants contribute the most to a nation’s carbon emissions. Moreover, a nation can have a relatively low disproportionality Gini coefficient, but still possess a substantially large carbon footprint due to the majority of its power plants burning large quantities of fossil-fuels and inefficiently producing electricity. Much more research is needed to explain such cases, if they exist, as well as how carbon emissions are distributed unevenly at the subnational level. We hope the findings for this study will provide a useful starting point for such future investigations.

## Methods

To calculate the disproportionality Gini coefficients for the 161 nations in the study, we used the “egen_inequal” module in Stata software (Version 13) and annual facility-level measures of carbon emissions for 19, 941 fossil-fuel power plants located in one of 161 nations for the year 2009 (the most recent year for which these data are currently available). Stata’s “egen_inequal” module provides a suite of programs for calculating various inequality measures, including the Gini coefficient, and also allows for the weighting of cases by a specified variable in the calculation of Gini coefficients. In-depth descriptions of the programs in the module are available by conducting a topic search for “egen-inequal” in the help section of Stata software (Version 8 and newer).

The emissions data measure the total pounds of carbon dioxide emitted by a plant in the year 2009, and are obtained from the Center for Global Development’s Carbon Monitoring for Action (CARMA) data file. CARMA draws on 3 data sets: plant-level emissions reports from the United States, European Union, Canada, and India; global plant- and company-level data from Platt’s World Electric Power Plants Database; and country-specific power production data from the U.S. Energy Information Agency. For non-reporting plants, CARMA estimates emissions using a statistical model fitted to data for the reporting plants and detailed data from the other 2 sources on plant-level engineering specifications. According to the creators of the CARMA data set^18^, for any given plant in CARMA, it is estimated that the reported value is within 20 percent of the actual value in 75 percent of cases for carbon dioxide emission levels.

For the calculation of the national Gini coefficients we weighted each fossil-fuel power plant by plant-level output, measured as net megawatt hours generated in 2009. These plant-level data are obtained from the Platts World Electric Power Plants Database. For the 19,941 power plants in the dataset, the Pearson’s correlation coefficient for plant-level output and plant size (measured as megawatt capacity) is 0.90 (0.001 level of statistical significance, two-tailed test), suggestive of inefficiencies among some of the larger power plants, which justifies the analysis of the national disproportionality Gini coefficients with plants weighted by plant-level output instead of by plant size.

To estimate the regression models, we used ordinary least squares (OLS) regression and robust regression, a combined approach suggested by researchers when analyzing cross-sectional data for nations^19^. OLS is among the most common regression methods used across the social and natural sciences. In the OLS models we estimate jackknife standard errors, which require no assumptions about underlying distributions. Robust regression is a relatively conservative approach that down-weights the influence of outliers in residuals^14^. In the robust regression models we employed a biweight tuning constant of 7, meaning seven times the median absolute deviation from the median residual, which is the default in Stata software (Version 13). The results were substantively the same if we moderately increased or decreased the value of the biweight tuning constant.

Since robust regression down-weights the influence of cases based on the size of their error terms, the R-squared statistic cannot be calculated for such models. As a reminder, all non-binary variables were converted into logarithmic form (base 10), leading to the estimation of elasticity coefficients, an approach well–established by prior research on the human drivers of carbon emissions and other related outcomes^9^,^12^,^13^.

The dependent variable in the regression analysis is national-level carbon emissions from fossil-fuel power plants for the year 2009. To create these national-level measures for the 161 nations in the study, using the CARMA data, we summed all plant-level emissions for the fossil-fuel power plants within each nation. As a validity check, we compared these summed values with the International Energy Agency’s (IEA) 2009 annual national measures of carbon dioxide emissions from fossil fuel combustion for the electivity and heat production sector. These data are readily available in the 2011 online edition of the IEA Statistics’ *Report on CO*_*2*_ *Emissions from Fuel Combustion*.

For the 121 nations that overlap between the two datasets (due to missing data in the IEA’s dataset), the Pearson’s correlation for our summed measure of national carbon emissions from fossil-fuel power plants and the IEA’s measure is 0.99 (0.001 level of statistical significance, two-tailed test). We note that the results of unreported regression models where we instead used the IEA data as our dependent variable (which reduces the sample to 121 nations) are substantively identical to the reported findings concerning the positive effect of the disproportionality Gini coefficient on national emissions for the 161 nations included in the study.

All reported regression models include total population size and Gross Domestic Product (GDP) per capita as control variables. These data were obtained from the World Bank’s online World Development Indicators Database. Total population is based on the de facto definition, which counts all residents regardless of legal status or citizenship, except for refugees not permanently settled in the country of asylum, who are generally considered part of the population of their country of origin. The per capita GDP data are measured in constant 2005 U.S. dollars. Population size and GDP per capita are the two most commonly employed predictors in prior studies of national anthropogenic carbon emissions^9^. These as well as all other control variables included in the analysis are annual measures for the year 2009.

The majority of estimated regression models also control for the number of fossil-fuel power plants within each nation, whether or not a nation is located in a tropical climate, and the average price of electricity (measured in U.S. Dollars) for each nation. We used the CARMA plant-level data to identify the number of plants within each nation. To control for climate conditions, we created a dummy variable for tropical climate (coded 1 if a country’s predominant latitude is less than 30 from the equator)^20^. The average price of electricity data are obtained from the International Energy Agency’s online Energy Prices and Taxes Database.

Some of the regression models also include measures of the percent of nations’ fossil-fuel power plants whose primary fuel source is (1) coal, (2) gas fossil-fuels, and (3) liquid fossil-fuels. These three variables are labeled in reported tables as “percent coal fossil-fuel plants”, “percent gas fossil-fuel plants”, and “percent liquid fossil-fuel plants”. To create these national measures, we used data available from PLATTS, which identify what the primary fuel source is for each plant. The most fully saturated regression models also control for nations’ international trade as a percent of GDP^10^,^21^. These data are obtained from the World Bank’s online World Development Indicators Database.

As an additional robustness check, in unreported regression models we also control for urban population as percent of total population, which we obtained from the World Bank’s online World Development Indicators Database. The effect of urban population (and urbanization more broadly) is commonly considered in research on the human drivers of national greenhouse gas emissions^9^. In the unreported models, the estimated effect of urban population as percent of total population on national emissions from fossil-fuel power plants is nonsignificant, and its inclusion does not change the estimated effect of the disproportionality Gini coefficient. The nonsignificant effect of the urban population measure could be, at least partly, due to the analysis of carbon emissions from only nations’ fossil-fuel power plants instead of for all fossil-fuel burning activities^22^.

## Additional Information

**How to cite this article**: Jorgenson, A. *et al*. Disproportionality in Power Plants’ Carbon Emissions: A Cross-National Study. *Sci. Rep.* **6**, 28661; doi: 10.1038/srep28661 (2016).

## Supplementary Material

Supplementary Information

## Figures and Tables

**Table 1:** Gini Coefficients for Disproportionality in Plant-Level Carbon Emissions for the 20 Nations with Highest Overall Carbon Emissions from Fossil-Fuel Power Plants, 2009.

Nation	Disproportionality Gini Coefficient	Number of Fossil-Fuel Power Plants	Percent Coal Fossil-Fuel Power Plants	Percent Gas Fossil-Fuel Power Plants	Percent Liquid Fossil-Fuel Power Plants
China	35.87	1130	81.50	8.68	9.82
United States	48.86	2612	21.75	53.52	24.73
India	46.97	737	37.72	18.86	43.42
Russia	49.46	529	19.28	65.97	14.75
Japan	42.23	1908	4.61	38.63	56.76
Germany	58.17	965	12.23	71.71	16.06
South Korea	39.61	198	15.15	33.33	51.52
Australia	41.53	434	9.45	47.24	43.32
United Kingdom	50.55	782	3.07	79.41	17.52
Saudi Arabia	49.74	208	0.00	15.38	84.62
Poland	54.72	287	79.79	17.77	2.44
Italy	53.81	536	3.91	65.49	30.60
Iran	38.51	140	0.00	45.00	55.00
Mexico	45.84	177	2.26	46.33	51.41
Indonesia	58.15	533	6.75	12.57	80.68
Canada	50.91	449	4.23	45.43	50.33
Turkey	38.05	304	15.79	51.64	32.57
Spain	40.97	485	3.92	66.18	29.90
Kazakhstan	50.68	51	52.94	29.41	17.65
Thailand	45.03	92	15.22	54.35	30.43

**Table 2:** Cross-Sectional Elasticity Models of National-Level Carbon Emissions from Fossil-Fuel Power Plants, 2009 (Models 1–4).

	Model 1 OLS Jacknife	Model 1 Robust Regression	Model 2 OLS Jacknife	Model 2 Robust Regression	Model 3 OLS Jacknife	Model 3 Robust Regression	Model 4 OLS Jacknife	Model 4 Robust Regression
Gini Coefficient for Disproportionality	.65***(.22)	.37**(.21)	.61**(.25)	.43**(.25)	.59***(.24)	.45**(.25)	.56**(.24)	.41**(.24)
Population Size	.93***(.05)	.99***(.05)	.92***(.07)	1.01***(.08)	.85***(.08)	.93***(.08)	.82***(.08)	.91***(.08)
GDP Per Capita	.99***(.07)	.96***(.06)	.98***(.08)	.98***(.07)	.86***(.09)	.86***(.08)	.85***(.09)	.85***(.08)
Number of Fossil-Fuel Power Plants			.03(.12)	−.05(.12)	.06(.12)	−.01(.12)	.09(.12)	−.01(.12)
Tropical Climate					−.27***(.10)	−.26***(.09)	−.26**(.10)	−.24***(.09)
Price of Electricity							−3.67**(1.75)	−3.56**(1.76)
R-squared	.79		.79		.80		.80	

Notes: all variables except “Tropical Climate” are in base 10 logarithmic form; ***p < 0.01 **p < 0.05 *p < 0.10 (one-tailed tests); biweight tuning constant is 7 in robust regression models; N = 161 Nations.

**Table 3:** Cross-Sectional Elasticity Models of National-Level Carbon Emissions from Fossil-Fuel Power Plants, 2009 (Models 5–7).

	Model 5 OLS Jacknife	Model 5 Robust Regression	Model 6 OLS Jacknife	Model 6 Robust Regression	Model 7 OLS Jacknife	Model 7 Robust Regression
Gini Coefficient for Disproportionality	.54**(.23)	.39**(.23)	.55**(.24)	.39**(.23)	.70***(.21)	.47**(.20)
Population Size	.68***(.09)	.78***(.09)	.70***(.10)	.78***(.09)	.80***(.05)	.85***(.05)
GDP Per Capita	.73***(.10)	.71***(.09)	.71***(.11)	.70***(.09)	.88***(.07)	.86***(.06)
Number of Fossil-Fuel Power Plants	.16*(.12)	.09(.11)	.18*(.13)	.10(.12)		
Tropical Climate	−.05(.12)	−.02(.10)	−.08(.12)	−.07(.10)		
Price of Electricity	−2.84**(1.49)	−2.97**(1.67)	−2.87**(1.54)	−2.96**(1.67)	−2.84**(1.55)	−3.09**(1.67)
Percent Coal Fossil-Fuel Power Plants	.32***(.07)	.29***(.07)	.31***(.08)	.27***(.07)	.36***(.06)	.32***(.07)
Percent Gas Fossil-Fuel Plants	.08(.11)	.14**(.08)	.08(.11)	.12*(.09)		
Percent Liquid Fossil-Fuel Plants	−.10(.12)	−.05(.10)	−.08(.12)	−.04(.11)		
Trade as Percent of GDP			.31*(.22)	.33*(.23)		
R-squared	.83		.83		.82	

Notes: all variables except “Tropical Climate” are in base 10 logarithmic form; ***p < 0.01 **p < 0.05 *p < 0.10 (one-tailed tests); biweight tuning constant is 7 in robust regression models; N = 161 Nations.
