# Supplementary material for: Disproportionality in Power Plants’ Carbon Emissions: A Cross-National Study
Source: Sci Rep. 2016 Jul 1;6:28661. doi: 10.1038/srep28661 (PMC4929454; doi:10.1038/srep28661)
Supplement: Supplementary Information [file srep28661-s1.pdf]

# Disproportionality in Power Plants' Carbon Emissions: A Cross-National Study

**Andrew Jorgenson [1,*], Wesley Longhofer [2], Don Grant [3]**

[1] Department of Sociology and the Environmental Studies Program, Boston College, 140 Commonwealth Avenue, Chestnut Hill, MA 02467 USA.

[2] Goizueta Business School, Emory University, 1300 Clifton Road, Atlanta, GA 30322 USA.

[3] Department of Sociology and the Renewable and Sustainable Energy Institute, University of Colorado at Boulder, Boulder, CO 80309 USA.

[*] corresponding author: jorgenan@bc.edu

**Supplementary Table 1. National Gini Coefficients for Disproportionality in Plant-Level Carbon Emissions**

| Nation | Disproportionality Gini Coefficient | Number of Fossil-Fuel Power Plants | Percent Coal Fossil-Fuel Power Plants | Percent Gas Fossil-Fuel Power Plants | Percent Liquid Fossil-Fuel Power Plants |
|---|---|---|---|---|---|
| Afghanistan | 38.47 | 32 | 0.00 | 9.38 | 90.63 |
| Albania | 14.52 | 4 | 0.00 | 0.00 | 100.00 |
| Algeria | 39.22 | 59 | 0.00 | 71.19 | 28.81 |
| Antigua and Barbuda | 24.50 | 6 | 0.00 | 0.00 | 100.00 |
| Argentina | 33.78 | 398 | 0.25 | 15.08 | 84.67 |
| Armenia | 5.48 | 2 | 0.00 | 100.00 | 0.00 |
| Australia | 41.53 | 434 | 9.45 | 47.24 | 43.32 |
| Austria | 38.20 | 78 | 5.13 | 73.08 | 21.80 |
| Azerbaijan | 32.97 | 16 | 0.00 | 87.50 | 12.50 |
| Bahamas | 30.83 | 30 | 0.00 | 0.00 | 100.00 |
| Bahrain | 21.61 | 12 | 0.00 | 100.00 | 0.00 |
| Bangladesh | 37.59 | 105 | 0.95 | 60.95 | 38.10 |
| Barbados | 18.72 | 4 | 0.00 | 0.00 | 100.00 |
| Belarus | 35.84 | 32 | 0.00 | 93.75 | 6.25 |
| Belgium | 37.75 | 196 | 5.61 | 71.43 | 22.96 |
| Belize | 23.68 | 8 | 0.00 | 0.00 | 100.00 |
| Benin | 36.39 | 12 | 0.00 | 0.00 | 100.00 |
| Bermuda | 17.31 | 3 | 0.00 | 0.00 | 100.00 |
| Bolivia | 30.11 | 16 | 0.00 | 50.00 | 50.00 |
| Bosnia and Herzegovina | 24.10 | 16 | 81.25 | 0.00 | 18.75 |
| Brazil | 52.61 | 157 | 3.82 | 31.21 | 64.97 |
| Brunei Darussalam | 36.54 | 10 | 0.00 | 80.00 | 20.00 |
| Bulgaria | 41.93 | 27 | 40.74 | 59.26 | 0.00 |
| Burkina Faso | 29.50 | 41 | 0.00 | 0.00 | 100.00 |
| Cambodia | 27.72 | 32 | 0.00 | 0.00 | 100.00 |
| Cameroon | 28.13 | 10 | 0.00 | 0.00 | 100.00 |
| Canada | 50.91 | 449 | 4.23 | 45.43 | 50.33 |
| Central African Republic | 13.95 | 15 | 0.00 | 0.00 | 100.00 |
| Chad | 26.53 | 13 | 0.00 | 0.00 | 100.00 |
| Chile | 32.87 | 60 | 16.67 | 13.33 | 70.00 |
| China | 35.87 | 1130 | 81.50 | 8.67 | 9.82 |
| Colombia | 36.20 | 65 | 9.23 | 43.08 | 47.69 |
| Comoros | 23.29 | 10 | 0.00 | 0.00 | 100.00 |
| Congo, Dem. Rep. | 15.28 | 35 | 0.00 | 2.86 | 97.14 |
| Congo, Rep. | 18.71 | 3 | 0.00 | 100.00 | 0.00 |
| Costa Rica | 27.69 | 7 | 0.00 | 0.00 | 100.00 |
| Cote d'Ivoire | 19.39 | 6 | 0.00 | 66.67 | 33.33 |
| Croatia | 33.16 | 18 | 5.56 | 27.78 | 66.67 |
| Cuba | 41.09 | 48 | 0.00 | 6.25 | 93.75 |
| Cyprus | 13.88 | 8 | 0.00 | 0.00 | 100.00 |

| | | | | | |
|---|---|---|---|---|---|
| Czech Republic | 42.55 | 74 | 44.60 | 54.05 | 1.35 |
| Denmark | 35.07 | 367 | 1.09 | 94.01 | 4.91 |
| Djibouti | 12.55 | 5 | 0.00 | 0.00 | 100.00 |
| Dominican Republic | 51.07 | 52 | 3.85 | 3.85 | 92.31 |
| Ecuador | 38.80 | 90 | 0.00 | 8.89 | 91.11 |
| Egypt | 31.73 | 78 | 0.00 | 38.46 | 61.54 |
| El Salvador | 34.45 | 12 | 0.00 | 0.00 | 100.00 |
| Equatorial Guinea | 4.85 | 2 | 0.00 | 0.00 | 100.00 |
| Eritrea | 14.93 | 6 | 0.00 | 0.00 | 100.00 |
| Estonia | 18.02 | 12 | 58.33 | 41.67 | 0.00 |
| Ethiopia | 34.19 | 44 | 0.00 | 0.00 | 100.00 |
| Fiji | 35.01 | 17 | 0.00 | 0.00 | 100.00 |
| Finland | 44.23 | 79 | 17.72 | 36.71 | 45.57 |
| France | 48.14 | 493 | 7.91 | 70.59 | 21.50 |
| Gabon | 37.91 | 31 | 0.00 | 9.68 | 90.32 |
| Gambia | 20.16 | 8 | 0.00 | 0.00 | 100.00 |
| Georgia | 11.62 | 6 | 0.00 | 66.67 | 33.33 |
| Germany | 58.17 | 965 | 12.23 | 71.71 | 16.06 |
| Ghana | 13.07 | 9 | 0.00 | 0.00 | 100.00 |
| Greece | 41.93 | 84 | 7.14 | 34.52 | 58.33 |
| Greenland | 39.36 | 21 | 0.00 | 0.00 | 100.00 |
| Guatemala | 31.27 | 15 | 6.67 | 0.00 | 93.33 |
| Guinea | 25.18 | 13 | 0.00 | 0.00 | 100.00 |
| Guyana | 26.02 | 19 | 0.00 | 0.00 | 100.00 |
| Haiti | 26.60 | 13 | 0.00 | 0.00 | 100.00 |
| Honduras | 30.07 | 26 | 0.00 | 0.00 | 100.00 |
| Hong Kong | 17.22 | 8 | 25.00 | 12.50 | 62.50 |
| Hungary | 52.29 | 68 | 8.82 | 82.35 | 8.82 |
| India | 46.97 | 737 | 37.72 | 18.86 | 43.42 |
| Indonesia | 58.15 | 533 | 6.75 | 12.57 | 80.68 |
| Iran | 38.51 | 140 | 0.00 | 45.00 | 55.00 |
| Iraq | 32.04 | 23 | 0.00 | 0.00 | 100.00 |
| Ireland | 40.25 | 38 | 2.63 | 78.95 | 18.42 |
| Israel | 33.46 | 25 | 12.00 | 36.00 | 52.00 |
| Italy | 53.81 | 536 | 3.92 | 65.49 | 30.60 |
| Japan | 42.23 | 1908 | 4.61 | 38.63 | 56.76 |
| Jordan | 21.53 | 23 | 0.00 | 21.74 | 78.26 |
| Kazakhstan | 50.68 | 51 | 52.94 | 29.41 | 17.65 |
| Kenya | 24.72 | 21 | 0.00 | 0.00 | 100.00 |
| Kiribati | 23.11 | 4 | 0.00 | 0.00 | 100.00 |
| Kuwait | 30.41 | 19 | 0.00 | 26.32 | 73.68 |
| Kyrgyz Republic | 9.96 | 3 | 33.33 | 66.67 | 0.00 |
| Latvia | 19.78 | 29 | 0.00 | 82.76 | 17.24 |
| Lebanon | 26.13 | 18 | 0.00 | 0.00 | 100.00 |

| | | | | | |
|---|---|---|---|---|---|
| Liberia | 12.81 | 3 | 0.00 | 0.00 | 100.00 |
| Libya | 34.07 | 58 | 0.00 | 10.35 | 89.66 |
| Lithuania | 22.06 | 9 | 0.00 | 77.78 | 22.22 |
| Luxembourg | 11.29 | 57 | 0.00 | 100.00 | 0.00 |
| Macau | 7.19 | 4 | 0.00 | 0.00 | 100.00 |
| Macedonia | 15.38 | 6 | 33.33 | 33.33 | 33.33 |
| Madagascar | 39.29 | 116 | 0.00 | 0.00 | 100.00 |
| Malawi | 18.25 | 9 | 0.00 | 0.00 | 100.00 |
| Malaysia | 40.04 | 161 | 3.73 | 23.60 | 72.67 |
| Maldives | 53.22 | 189 | 0.00 | 0.00 | 100.00 |
| Mali | 36.22 | 21 | 0.00 | 0.00 | 100.00 |
| Mauritania | 21.71 | 27 | 0.00 | 0.00 | 100.00 |
| Mauritius | 24.65 | 9 | 33.33 | 0.00 | 66.67 |
| Mexico | 45.84 | 177 | 2.26 | 46.33 | 51.41 |
| Moldova | 31.17 | 13 | 0.00 | 100.00 | 0.00 |
| Mongolia | 22.11 | 25 | 32.00 | 0.00 | 68.00 |
| Morocco | 41.18 | 18 | 16.67 | 11.11 | 72.22 |
| Mozambique | 15.72 | 8 | 0.00 | 25.00 | 75.00 |
| Namibia | 2.85 | 3 | 33.33 | 0.00 | 66.67 |
| Netherlands | 48.79 | 591 | 0.51 | 98.14 | 1.35 |
| New Zealand | 38.11 | 47 | 6.38 | 70.21 | 23.40 |
| Nicaragua | 22.56 | 26 | 0.00 | 0.00 | 100.00 |
| Niger | 40.95 | 11 | 9.09 | 0.00 | 90.91 |
| Nigeria | 37.09 | 106 | 0.00 | 35.85 | 64.15 |
| Norway | 22.55 | 20 | 5.00 | 45.00 | 50.00 |
| Oman | 38.56 | 98 | 0.00 | 28.57 | 71.43 |
| Pakistan | 42.58 | 117 | 0.86 | 32.48 | 66.67 |
| Panama | 29.29 | 80 | 0.00 | 0.00 | 100.00 |
| Papua New Guinea | 25.20 | 34 | 0.00 | 5.88 | 94.12 |
| Peru | 37.43 | 285 | 0.35 | 3.86 | 95.79 |
| Philippines | 34.44 | 281 | 4.27 | 2.49 | 93.24 |
| Poland | 54.72 | 287 | 79.79 | 17.77 | 2.44 |
| Portugal | 44.82 | 190 | 1.05 | 45.79 | 53.16 |
| Puerto Rico | 33.62 | 20 | 5.00 | 5.00 | 90.00 |
| Qatar | 30.42 | 15 | 0.00 | 100.00 | 0.00 |
| Romania | 45.31 | 53 | 35.85 | 52.83 | 11.32 |
| Russia | 49.46 | 529 | 19.28 | 65.97 | 14.75 |
| Saint Kitts and Nevis | 12.09 | 3 | 0.00 | 0.00 | 100.00 |
| Saint Lucia | 9.40 | 3 | 0.00 | 0.00 | 100.00 |
| Saint Vincent | 16.67 | 6 | 0.00 | 0.00 | 100.00 |
| Samoa | 18.65 | 4 | 0.00 | 0.00 | 100.00 |
| Sao Tome and Principe | 4.82 | 4 | 0.00 | 0.00 | 100.00 |
| Saudi Arabia | 49.74 | 208 | 0.00 | 15.39 | 84.62 |
| Senegal | 39.66 | 53 | 0.00 | 0.00 | 100.00 |

| | | | | | |
|---|---|---|---|---|---|
| Serbia | 22.98 | 22 | 45.46 | 9.09 | 45.46 |
| Sierra Leone | 29.77 | 16 | 0.00 | 0.00 | 100.00 |
| Singapore | 20.90 | 40 | 0.00 | 37.50 | 62.50 |
| Slovak Republic | 41.70 | 36 | 41.67 | 30.56 | 27.78 |
| Slovenia | 21.82 | 23 | 21.74 | 30.44 | 47.83 |
| Solomon Islands | 33.94 | 11 | 0.00 | 0.00 | 100.00 |
| South Korea | 39.61 | 198 | 15.15 | 33.33 | 51.52 |
| Spain | 40.97 | 485 | 3.92 | 66.19 | 29.90 |
| Sri Lanka | 31.98 | 25 | 0.00 | 0.00 | 100.00 |
| Sudan | 30.86 | 37 | 0.00 | 0.00 | 100.00 |
| Suriname | 19.08 | 14 | 0.00 | 0.00 | 100.00 |
| Sweden | 34.77 | 49 | 6.12 | 44.90 | 48.98 |
| Tanzania | 9.30 | 37 | 2.70 | 8.11 | 89.19 |
| Thailand | 45.03 | 92 | 15.22 | 54.35 | 30.44 |
| Togo | 18.55 | 4 | 0.00 | 0.00 | 100.00 |
| Tonga | 9.89 | 4 | 0.00 | 0.00 | 100.00 |
| Trinidad and Tobago | 20.31 | 7 | 0.00 | 85.71 | 14.29 |
| Tunisia | 29.78 | 20 | 0.00 | 75.00 | 25.00 |
| Turkey | 38.05 | 304 | 15.79 | 51.65 | 32.57 |
| Turkmenistan | 35.06 | 10 | 0.00 | 100.00 | 0.00 |
| Uganda | 7.27 | 13 | 0.00 | 0.00 | 100.00 |
| Ukraine | 29.14 | 48 | 29.17 | 60.42 | 10.42 |
| United Arab Emirates | 33.83 | 71 | 0.00 | 70.42 | 29.58 |
| United Kingdom | 50.55 | 782 | 3.07 | 79.41 | 17.52 |
| United States | 48.86 | 2612 | 21.75 | 53.52 | 24.73 |
| Uruguay | 14.38 | 3 | 0.00 | 0.00 | 100.00 |
| Uzbekistan | 30.14 | 10 | 20.00 | 70.00 | 10.00 |
| Vanuatu | 23.09 | 5 | 0.00 | 0.00 | 100.00 |
| Venezuela | 46.67 | 51 | 0.00 | 68.63 | 31.37 |
| Vietnam | 28.68 | 45 | 28.89 | 20.00 | 51.11 |
| Yemen | 33.92 | 31 | 0.00 | 0.00 | 100.00 |
| Zambia | 15.55 | 14 | 0.00 | 0.00 | 100.00 |
| Zimbabwe | 17.38 | 6 | 66.67 | 0.00 | 33.33 |
